# Aqua­tricarbon­yl(4-carboxy­pyridine-2-carboxyl­ato-κ^2^ *N*,*O* ^2^)rhenium(I)

**DOI:** 10.1107/S160053680802761X

**Published:** 2008-09-06

**Authors:** Marietjie Schutte, Hendrik G. Visser

**Affiliations:** a Department of Chemistry, University of the Free State, PO Box 339, Bloemfontein 9300, South Africa

## Abstract

There are two mol­ecules with similar bond dimensions in the asymmetric unit of the title complex, [Re(C_7_H_4_NO_4_)(CO)_3_(H_2_O)]. The metal centre is coordinated facially by three carbonyl groups, is chelated by a 4-carboxy­pyridine-2-carboxyl­ate ligand and is also coordinated by a water mol­ecule. O—H⋯O hydrogen bonds give rise to a three-dimensional network.

## Related literature

For the monoclinic polymorph of the title compound, see: Mundwiler *et al.* (2004 ▶). For related structures, see: Kemp (2006 ▶); Roodt *et al.* (2003 ▶); Schutte *et al.* (2007 ▶); Wang *et al.* (2003 ▶); Alvarez *et al.* (2007 ▶). For the synthesis of the precursor, see: Alberto *et al.* (1996 ▶);
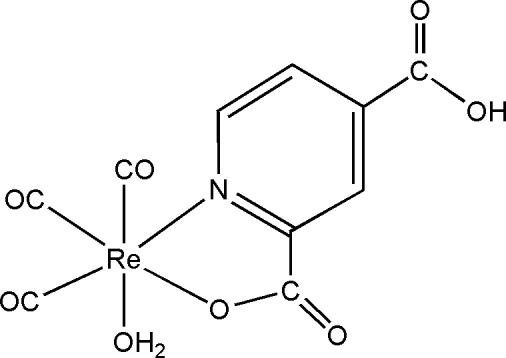


## Experimental

### 

#### Crystal data

[Re(C_7_H_4_NO_4_)(CO)_3_(H_2_O)]
*M* *_r_* = 454.37
Triclinic, 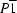

*a* = 9.5024 (11) Å
*b* = 12.4254 (16) Å
*c* = 12.4889 (16) Å
α = 101.799 (4)°
β = 107.943 (4)°
γ = 111.346 (4)°
*V* = 1220.4 (3) Å^3^
*Z* = 4
Mo *K*α radiation
μ = 10.00 mm^−1^
*T* = 100 (2) K
0.27 × 0.17 × 0.05 mm

#### Data collection

Bruker APEXII diffractometer
Absorption correction: multi-scan (*SADABS*; Bruker, 2004 ▶) *T*_min_ = 0.140, *T*_max_ = 0.605
15096 measured reflections
5848 independent reflections
4869 reflections with *I* > 2σ(*I*)
*R*_int_ = 0.037

#### Refinement

*R*[*F* ^2^ > 2σ(*F* ^2^)] = 0.026
*wR*(*F* ^2^) = 0.065
*S* = 1.05
5848 reflections
373 parameters
7 restraints
H atoms treated by a mixture of independent and constrained refinement
Δρ_max_ = 1.21 e Å^−3^
Δρ_min_ = −1.80 e Å^−3^

### 

Data collection: *APEX2* (Bruker, 2005 ▶); cell refinement: *SAINT-Plus* (Bruker, 2004 ▶); data reduction: *SAINT-Plus*; program(s) used to solve structure: *SHELXS97* (Sheldrick, 2008 ▶); program(s) used to refine structure: *SHELXL97* (Sheldrick, 2008 ▶); molecular graphics: *DIAMOND* (Brandenberg & Putz, 2005 ▶) and *ORTEP-3* (Farrugia, 1999 ▶); software used to prepare material for publication: *SHELXL97*.

## Supplementary Material

Crystal structure: contains datablocks global, I. DOI: 10.1107/S160053680802761X/ng2479sup1.cif

Structure factors: contains datablocks I. DOI: 10.1107/S160053680802761X/ng2479Isup2.hkl

Additional supplementary materials:  crystallographic information; 3D view; checkCIF report

## Figures and Tables

**Table:** 

N1—Re1	2.180 (4)
O5—Re1	2.153 (3)
O15—Re2	2.148 (3)
N2—Re2	2.166 (4)
C11—Re2	1.892 (5)
C12—Re2	1.947 (5)
C2—Re1	1.906 (5)
C3—Re1	1.885 (6)
C13—Re2	1.883 (5)
O14—Re2	2.153 (3)
O4—Re1	2.170 (3)
Re1—C1	1.915 (5)

**Table:** 

C11—Re2—C12	89.52 (19)
C11—Re2—O15	170.52 (16)
C12—Re2—O15	98.45 (16)
C11—Re2—O14	95.63 (16)
C12—Re2—O14	96.35 (17)
O15—Re2—O14	78.48 (12)
O15—Re2—N2	74.98 (12)
C2—Re1—O5	98.77 (15)
C1—Re1—O4	95.49 (17)
O5—Re1—O4	80.73 (12)
O5—Re1—N1	74.77 (12)

**Table 2:** Hydrogen-bond geometry (Å, °)

*D*—H⋯*A*	*D*—H	H⋯*A*	*D*⋯*A*	*D*—H⋯*A*
O7—H7⋯O15^i^	0.82	1.81	2.595 (4)	160
O4—H4*B*⋯O16^ii^	0.85 (5)	2.51 (5)	2.920 (5)	111 (4)
O4—H4*B*⋯O8^iii^	0.85 (5)	2.01 (3)	2.780 (5)	150 (5)
O4—H4*B*⋯O16^ii^	0.85 (5)	2.51 (5)	2.920 (5)	111 (4)
O14—H14*B*⋯O6	0.85 (5)	1.84 (2)	2.671 (5)	169 (5)
O14—H14*A*⋯O18^iii^	0.840 (18)	1.87 (2)	2.674 (4)	161 (5)

Symmetry codes: (i) 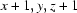; (ii) 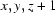; (iii) 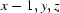.

**Fig. 1.**
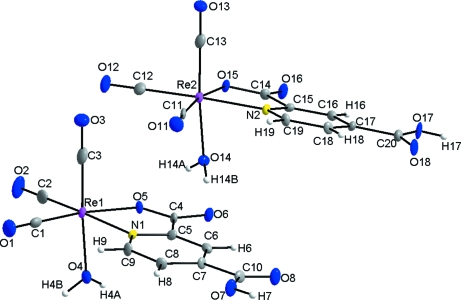
Representation of the title compound, showing the numbering scheme and displacement ellipsoids (50% probability).

## supplementary crystallographic information

### Comment

The title compound, Re(C_10_H_6_NO_8_),is one of many Re(I)-tricarbonyl complexes currently under investigation in the field of radiopharmacology. One polymorph of the title compound was reported earlier [Mundwiler *et al.*, (2004)].

The Re(I) core is coordinated by three facial carbonyl groups, one pyridine-2-carboxylato-4-carboxylic acid ligand and a water molecule. A slightly distorted octahedral geometry around the Re(I) metal centre is observed, possibly due to the effect of the small bite angles of 74.78 (14)° and 74.98 (12)° respectively for the two pyridine-2,4-dicarboxylic acid units. The Re—OH_2_ bond distances of 2.153 (4) Å and 2.170 (4) Å compare well with related structures [Mundwiler *et al.*, (2004) and Kemp, (2006)] of 2.198 (5) Å and 2.192 (4) Å. The Re—CO distances are well within the normal range, 1.883 (6) Å to 1.947 (6) Å. The crystal structure shows a range of hydrogen bonding of the types OH—O and CH—O thereby forming a 3D polymeric network, with DA distances ranging from 2.595 (4) Å to 3.426 (5) Å.

### Experimental

[NEt_4_]_2_[Re(CO)_3_Br_3_] (300 mg, 0.389 mmol), as prepared by Alberto *et al.* (1996) was stirred in 40 ml of water at pH 2.2 for 20 minutes until dissolved. AgNO_3_ (198 mg, 1.167 mmol) was added to the solution and stirred for 24 h at room temperature. The precipitate, AgBr, was filtered off and weighed(0.220 g). 2,4-Pyridinedicarboxylic acid (65 mg, 0.389 mmol) was added to the filtrate as a solid and stirred for 36 h. The solution turned bright yellow with a light yellow precipitate. The product was filtered off, dried and weighed. Crystals were obtained by slow evaporation of the filtrate. (Yield: 0.240 g, 68%).

### Refinement

The aromatic H atoms were placed in geometrically idealized positions and constrained to ride on its parent atoms with U<i/>_iso_(H) = 1.2U<i/>_eq_(C).  The highest electron density lies within 1.14 Å from Re1.  The hydrogen atoms of the coordinated water molecules were determined from a difference Fourier map and their positional parameters freely refined with U<i/>_iso_(H) = 1.5U<i/>_eq_(O).

### Crystal data

**Table:**

[Re(C_7_H_4_NO_4_)(CO)_3_(H_2_O)]	*Z* = 4
*M**_r_* = 454.37	*F*(000) = 848.0
Triclinic, *P*1	*D*_x_ = 2.473 Mg m^−^^3^
Hall symbol: -P 1	Mo *K*α radiation, λ = 0.71073 Å
*a* = 9.5024 (11) Å	Cell parameters from 7140 reflections
*b* = 12.4254 (16) Å	θ = 2.8–28.3°
*c* = 12.4889 (16) Å	µ = 10.00 mm^−^^1^
α = 101.799 (4)°	*T* = 100 K
β = 107.943 (4)°	Plate, yellow
γ = 111.346 (4)°	0.27 × 0.17 × 0.05 mm
*V* = 1220.4 (3) Å^3^	

### Data collection

**Table:**

Bruker APEX diffractometer	4869 reflections with *I* > 2σ(*I*)
φ and ω scans	*R*_int_ = 0.037
Absorption correction: multi-scan (SADABS; Bruker, 2004)	θ_max_ = 28.3°, θ_min_ = 1.8°
*T*_min_ = 0.140, *T*_max_ = 0.605	*h* = −12→12
15096 measured reflections	*k* = −16→15
5048 independent reflections	*l* = −16→16

### Refinement

**Table:**

Refinement on *F*^2^	7 restraints
Least-squares matrix: full	H atoms treated by a mixture of independent and constrained refinement
*R*[*F*^2^ > 2σ(*F*^2^)] = 0.027	*w* = 1/[σ^2^(*F*_o_^2^) + (0.0251*P*)^2^] where *P* = (*F*_o_^2^ + 2*F*_c_^2^)/3
*wR*(*F*^2^) = 0.065	(Δ/σ)_max_ = 0.015
*S* = 1.05	Δρ_max_ = 1.21 e Å^−^^3^
5848 reflections	Δρ_min_ = −1.80 e Å^−^^3^
373 parameters	

### Special details

**Table:**

Geometry. All e.s.d.'s (except the e.s.d. in the dihedral angle between two l.s. planes) are estimated using the full covariance matrix. The cell e.s.d.'s are taken into account individually in the estimation of e.s.d.'s in distances, angles and torsion angles; correlations between e.s.d.'s in cell parameters are only used when they are defined by crystal symmetry. An approximate (isotropic) treatment of cell e.s.d.'s is used for estimating e.s.d.'s involving l.s. planes.

### Fractional atomic coordinates and isotropic or equivalent isotropic displacement parameters (Å^2^)

**Table:**

	*x*	*y*	*z*	*U*_iso_*/*U*_eq_	
N1	0.3078 (4)	0.2654 (3)	0.5400 (3)	0.0095 (8)	
O5	0.1030 (4)	0.1933 (3)	0.3130 (2)	0.0113 (7)	
O6	0.2391 (4)	0.1072 (3)	0.2433 (3)	0.0152 (7)	
C4	0.2193 (6)	0.1635 (4)	0.3289 (4)	0.0116 (9)	
O15	0.0841 (4)	0.2152 (3)	−0.1450 (3)	0.0112 (7)	
C5	0.3392 (6)	0.1998 (4)	0.4572 (4)	0.0102 (9)	
O16	0.0735 (4)	0.0827 (3)	−0.3002 (3)	0.0160 (7)	
C15	0.3019 (5)	0.1585 (4)	−0.1099 (4)	0.0097 (9)	
C14	0.1407 (6)	0.1487 (4)	−0.1934 (4)	0.0118 (10)	
C18	0.5968 (5)	0.1949 (4)	0.0518 (4)	0.0116 (9)	
H18	0.6981	0.2089	0.1084	0.014*	
C17	0.5336 (6)	0.1168 (4)	−0.0662 (4)	0.0115 (9)	
C16	0.3816 (5)	0.0981 (4)	−0.1491 (4)	0.0106 (9)	
H16	0.3358	0.0459	−0.2288	0.013*	
C8	0.5409 (6)	0.2756 (4)	0.6944 (4)	0.0127 (10)	
H8	0.6081	0.3019	0.7761	0.015*	
C6	0.4696 (6)	0.1716 (4)	0.4875 (4)	0.0129 (10)	
H6	0.4884	0.1278	0.4282	0.015*	
C9	0.4082 (5)	0.3013 (4)	0.6568 (4)	0.0122 (10)	
H9	0.3874	0.3453	0.7146	0.015*	
C7	0.5727 (5)	0.2098 (4)	0.6085 (4)	0.0105 (9)	
C20	0.6310 (6)	0.0551 (4)	−0.0994 (4)	0.0131 (10)	
C19	0.5084 (5)	0.2516 (4)	0.0845 (4)	0.0118 (10)	
H19	0.5507	0.3031	0.1641	0.014*	
N2	0.3633 (4)	0.2346 (3)	0.0051 (3)	0.0092 (8)	
O8	0.7441 (4)	0.1233 (3)	0.5666 (3)	0.0200 (8)	
O7	0.8100 (4)	0.2290 (3)	0.7589 (3)	0.0190 (8)	
H7	0.8878	0.2118	0.773	0.029*	
C10	0.7178 (5)	0.1827 (4)	0.6423 (4)	0.0134 (10)	
O18	0.7570 (4)	0.0669 (3)	−0.0231 (3)	0.0174 (8)	
O17	0.5674 (4)	−0.0113 (3)	−0.2139 (3)	0.0176 (8)	
H17	0.6264	−0.0424	−0.2258	0.026*	
O11	0.4169 (4)	0.4220 (3)	0.3181 (3)	0.0206 (8)	
O12	−0.0486 (5)	0.3985 (3)	0.0753 (3)	0.0290 (9)	
C11	0.3339 (6)	0.3833 (4)	0.2154 (4)	0.0129 (10)	
C12	0.0473 (6)	0.3683 (4)	0.0652 (4)	0.0165 (10)	
O2	−0.2361 (5)	0.3030 (4)	0.3410 (3)	0.0296 (10)	
C2	−0.1119 (6)	0.3005 (4)	0.3895 (4)	0.0178 (11)	
C3	0.2069 (6)	0.4532 (5)	0.4629 (4)	0.0211 (12)	
O3	0.2698 (5)	0.5484 (3)	0.4535 (3)	0.0283 (9)	
C13	0.3283 (6)	0.4712 (5)	0.0407 (4)	0.0162 (10)	
O13	0.4026 (4)	0.5690 (3)	0.0395 (3)	0.0209 (8)	
O14	0.0791 (4)	0.1290 (3)	0.0380 (3)	0.0128 (7)	
O4	−0.0362 (4)	0.1106 (3)	0.4661 (3)	0.0191 (8)	
H4A	−0.070 (6)	0.046 (3)	0.408 (3)	0.029*	
H4B	−0.092 (6)	0.097 (4)	0.508 (4)	0.029*	
H14B	0.123 (5)	0.112 (5)	0.098 (3)	0.029*	
H14A	−0.025 (2)	0.093 (4)	0.010 (4)	0.029*	
Re2	0.20999 (2)	0.315032 (16)	0.046124 (15)	0.00973 (6)	
Re1	0.09158 (2)	0.296297 (16)	0.468442 (15)	0.01020 (6)	
C1	0.1002 (6)	0.3789 (5)	0.6194 (4)	0.0165 (10)	
O1	0.1138 (5)	0.4303 (3)	0.7126 (3)	0.0272 (9)	

### Atomic displacement parameters (Å^2^)

**Table:**

	*U*^11^	*U*^22^	*U*^33^	*U*^12^	*U*^13^	*U*^23^
N1	0.0096 (18)	0.013 (2)	0.0088 (18)	0.0082 (16)	0.0031 (15)	0.0057 (16)
O5	0.0128 (16)	0.0157 (18)	0.0070 (15)	0.0087 (14)	0.0052 (13)	0.0014 (13)
O6	0.0183 (17)	0.0185 (19)	0.0109 (16)	0.0125 (15)	0.0057 (14)	0.0022 (14)
C4	0.016 (2)	0.013 (2)	0.013 (2)	0.010 (2)	0.0097 (19)	0.0047 (19)
O15	0.0142 (16)	0.0144 (17)	0.0063 (14)	0.0101 (14)	0.0035 (13)	0.0007 (13)
C5	0.012 (2)	0.008 (2)	0.006 (2)	0.0038 (18)	0.0014 (17)	−0.0007 (17)
O16	0.0185 (18)	0.0204 (19)	0.0076 (15)	0.0131 (15)	0.0024 (13)	−0.0010 (14)
C15	0.012 (2)	0.008 (2)	0.009 (2)	0.0047 (18)	0.0040 (18)	0.0034 (18)
C14	0.012 (2)	0.016 (3)	0.010 (2)	0.008 (2)	0.0044 (18)	0.0060 (19)
C18	0.006 (2)	0.017 (3)	0.009 (2)	0.0058 (19)	0.0008 (17)	0.0032 (19)
C17	0.015 (2)	0.011 (2)	0.014 (2)	0.0092 (19)	0.0085 (19)	0.0059 (19)
C16	0.011 (2)	0.010 (2)	0.008 (2)	0.0039 (19)	0.0039 (17)	0.0016 (18)
C8	0.012 (2)	0.016 (2)	0.006 (2)	0.006 (2)	0.0017 (17)	0.0023 (18)
C6	0.013 (2)	0.010 (2)	0.013 (2)	0.0028 (19)	0.0073 (19)	0.0026 (19)
C9	0.013 (2)	0.015 (2)	0.006 (2)	0.008 (2)	0.0025 (18)	−0.0010 (18)
C7	0.005 (2)	0.014 (2)	0.011 (2)	0.0053 (18)	0.0015 (17)	0.0029 (19)
C20	0.012 (2)	0.014 (2)	0.016 (2)	0.008 (2)	0.0073 (19)	0.005 (2)
C19	0.010 (2)	0.014 (2)	0.008 (2)	0.0056 (19)	0.0030 (18)	0.0001 (18)
N2	0.0081 (18)	0.010 (2)	0.0085 (18)	0.0053 (16)	0.0021 (14)	0.0014 (15)
O8	0.0175 (18)	0.027 (2)	0.0152 (17)	0.0143 (16)	0.0046 (14)	0.0022 (15)
O7	0.0150 (17)	0.029 (2)	0.0117 (16)	0.0145 (16)	0.0008 (14)	0.0025 (15)
C10	0.006 (2)	0.017 (3)	0.016 (2)	0.0058 (19)	0.0029 (18)	0.006 (2)
O18	0.0116 (17)	0.026 (2)	0.0170 (17)	0.0135 (16)	0.0056 (14)	0.0040 (15)
O17	0.0187 (18)	0.025 (2)	0.0133 (17)	0.0179 (16)	0.0065 (14)	0.0014 (15)
O11	0.0230 (19)	0.021 (2)	0.0082 (16)	0.0068 (16)	0.0034 (14)	−0.0011 (15)
O12	0.028 (2)	0.028 (2)	0.041 (2)	0.0201 (19)	0.0204 (19)	0.0099 (19)
C11	0.013 (2)	0.012 (2)	0.019 (2)	0.009 (2)	0.0114 (19)	0.005 (2)
C12	0.018 (3)	0.011 (2)	0.018 (2)	0.006 (2)	0.007 (2)	0.002 (2)
O2	0.026 (2)	0.044 (3)	0.0178 (18)	0.027 (2)	0.0004 (16)	0.0021 (18)
C2	0.026 (3)	0.019 (3)	0.010 (2)	0.012 (2)	0.010 (2)	0.002 (2)
C3	0.022 (3)	0.020 (3)	0.018 (3)	0.012 (2)	0.007 (2)	−0.002 (2)
O3	0.040 (2)	0.020 (2)	0.033 (2)	0.0141 (19)	0.0231 (19)	0.0118 (18)
C13	0.018 (2)	0.016 (3)	0.012 (2)	0.010 (2)	0.0043 (19)	0.000 (2)
O13	0.029 (2)	0.0149 (19)	0.0237 (19)	0.0093 (16)	0.0168 (16)	0.0079 (15)
O14	0.0086 (16)	0.0141 (18)	0.0141 (17)	0.0046 (14)	0.0031 (13)	0.0054 (14)
O4	0.025 (2)	0.0138 (19)	0.0214 (19)	0.0088 (16)	0.0164 (16)	0.0019 (15)
Re2	0.00971 (10)	0.01041 (10)	0.00854 (9)	0.00554 (8)	0.00342 (7)	0.00128 (7)
Re1	0.01226 (10)	0.01220 (11)	0.00847 (9)	0.00860 (8)	0.00437 (7)	0.00248 (8)
C1	0.016 (2)	0.017 (3)	0.023 (3)	0.011 (2)	0.009 (2)	0.012 (2)
O1	0.039 (2)	0.028 (2)	0.0169 (19)	0.0175 (19)	0.0158 (17)	0.0020 (17)

### Geometric parameters (Å, °)

**Table:**

N1—C9	1.343 (5)	C7—C10	1.493 (6)
N1—C5	1.357 (5)	C20—O18	1.215 (5)
N1—Re1	2.180 (4)	C20—O17	1.309 (5)
O5—C4	1.261 (5)	C19—N2	1.337 (5)
O5—Re1	2.153 (3)	C19—H19	0.93
O6—C4	1.258 (5)	N2—Re2	2.166 (4)
C4—O6	1.258 (5)	O8—C10	1.223 (5)
C4—C5	1.508 (6)	O7—C10	1.313 (5)
O15—C14	1.287 (5)	O11—C11	1.161 (5)
O15—Re2	2.148 (3)	O12—C12	1.135 (6)
C5—C6	1.372 (6)	C11—Re2	1.892 (5)
O16—C14	1.227 (5)	C12—Re2	1.947 (5)
C15—N2	1.351 (5)	O2—C2	1.164 (6)
C15—C16	1.368 (6)	C2—Re1	1.906 (5)
C15—C14	1.508 (6)	C3—O3	1.165 (6)
C18—C19	1.376 (6)	C3—Re1	1.885 (6)
C18—C17	1.385 (6)	C13—O13	1.169 (6)
C18—H18	0.93	C13—Re2	1.883 (5)
C17—C16	1.395 (6)	O14—Re2	2.153 (3)
C17—C20	1.496 (6)	O14—H14B	0.85 (4)
C16—H16	0.93	O14—H14A	0.85 (4)
C8—C9	1.379 (6)	O4—Re1	2.170 (3)
C8—C7	1.390 (6)	O4—H4A	0.85 (5)
C8—H8	0.93	O4—H4B	0.85 (5)
C6—C7	1.388 (6)	Re1—C1	1.915 (5)
C6—H6	0.93	C1—O1	1.151 (5)
C9—H9	0.93		
			
C9—N1—C5	117.6 (4)	C15—N2—Re2	115.9 (3)
C9—N1—Re1	126.4 (3)	C10—O7—H7	109.5
C5—N1—Re1	115.9 (3)	O8—C10—O7	125.2 (4)
C4—O5—Re1	118.5 (3)	O8—C10—C7	121.8 (4)
O6—C4—O5	122.9 (4)	O7—C10—C7	113.0 (4)
O6—C4—O5	122.9 (4)	C20—O17—H17	109.5
O6—C4—C5	119.9 (4)	O11—C11—Re2	175.5 (4)
O6—C4—C5	119.9 (4)	O12—C12—Re2	179.4 (5)
O5—C4—C5	117.2 (4)	O2—C2—Re1	179.9 (5)
C14—O15—Re2	118.6 (3)	O3—C3—Re1	176.0 (5)
N1—C5—C6	123.1 (4)	O13—C13—Re2	178.8 (4)
N1—C5—C4	113.5 (4)	Re2—O14—H14B	116 (3)
C6—C5—C4	123.3 (4)	Re2—O14—H14A	121 (3)
N2—C15—C16	122.9 (4)	H14B—O14—H14A	112 (3)
N2—C15—C14	114.7 (4)	Re1—O4—H4A	124 (3)
C16—C15—C14	122.3 (4)	Re1—O4—H4B	121 (3)
O16—C14—O15	124.0 (4)	H4A—O4—H4B	110 (3)
O16—C14—C15	120.6 (4)	C13—Re2—C11	88.2 (2)
O15—C14—C15	115.4 (4)	C13—Re2—C12	87.6 (2)
C19—C18—C17	119.4 (4)	C11—Re2—C12	89.52 (19)
C19—C18—H18	120.3	C13—Re2—O15	97.19 (16)
C17—C18—H18	120.3	C11—Re2—O15	170.52 (16)
C18—C17—C16	118.9 (4)	C12—Re2—O15	98.45 (16)
C18—C17—C20	118.5 (4)	C13—Re2—O14	174.48 (16)
C16—C17—C20	122.6 (4)	C11—Re2—O14	95.63 (16)
C15—C16—C17	118.2 (4)	C12—Re2—O14	96.35 (17)
C15—C16—H16	120.9	O15—Re2—O14	78.48 (12)
C17—C16—H16	120.9	C13—Re2—N2	96.92 (17)
C9—C8—C7	119.0 (4)	C11—Re2—N2	96.70 (16)
C9—C8—H8	120.5	C12—Re2—N2	172.39 (16)
C7—C8—H8	120.5	O15—Re2—N2	74.98 (12)
C5—C6—C7	118.6 (4)	O14—Re2—N2	78.73 (13)
C5—C6—H6	120.7	C3—Re1—C2	88.6 (2)
C7—C6—H6	120.7	C3—Re1—C1	88.1 (2)
N1—C9—C8	122.7 (4)	C2—Re1—C1	88.6 (2)
N1—C9—H9	118.7	C3—Re1—O5	95.56 (17)
C8—C9—H9	118.7	C2—Re1—O5	98.77 (15)
C6—C7—C8	119.0 (4)	C1—Re1—O5	171.85 (16)
C6—C7—C10	119.0 (4)	C3—Re1—O4	176.21 (16)
C8—C7—C10	122.0 (4)	C2—Re1—O4	92.71 (18)
O18—C20—O17	124.8 (4)	C1—Re1—O4	95.49 (17)
O18—C20—C17	120.7 (4)	O5—Re1—O4	80.73 (12)
O17—C20—C17	114.5 (4)	C3—Re1—N1	96.75 (18)
N2—C19—C18	121.8 (4)	C2—Re1—N1	171.93 (16)
N2—C19—H19	119.1	C1—Re1—N1	97.60 (17)
C18—C19—H19	119.1	O5—Re1—N1	74.77 (12)
C19—N2—C15	118.7 (4)	O4—Re1—N1	81.58 (14)
C19—N2—Re2	125.3 (3)	O1—C1—Re1	176.6 (4)

### Hydrogen-bond geometry (Å, °)

**Table:**

*D*—H···*A*	*D*—H	H···*A*	*D*···*A*	*D*—H···*A*
O7—H7···O15^i^	0.82	1.81	2.595 (4)	160.
O4—H4B···O16^ii^	0.85 (5)	2.51 (5)	2.920 (5)	111 (4)
O4—H4B···O8^iii^	0.85 (5)	2.01 (3)	2.780 (5)	150 (5)
O4—H4B···O16^ii^	0.85 (5)	2.51 (5)	2.920 (5)	111 (4)
O14—H14B···O6	0.85 (5)	1.84 (2)	2.671 (5)	169 (5)
O14—H14A···O18^iii^	0.84 (2)	1.87 (2)	2.674 (4)	161 (5)
O17—H17···O6^iv^	0.82	1.78	2.602 (4)	177.
C8—H8···O13^v^	0.93	2.58	3.253 (5)	130.
C6—H6···O17^iv^	0.93	2.55	3.426 (5)	156.
C19—H19···O2^vi^	0.93	2.5	3.131 (5)	126.

Symmetry codes: (i) *x*+1, *y*, *z*+1; (ii) *x*, *y*, *z*+1; (iii) *x*−1, *y*, *z*;  (iv) −*x*+1, −*y*, −*z*; (v) −*x*+1, −*y*+1, −*z*+1; (vi) *x*+1, *y*, *z*.
